# An *Alcaligenes* strain emulates *Bacillus thuringiensis* producing a binary protein that kills corn rootworm through a mechanism similar to Cry34Ab1/Cry35Ab1

**DOI:** 10.1038/s41598-017-03544-9

**Published:** 2017-06-08

**Authors:** Nasser Yalpani, Dan Altier, Jennifer Barry, Adane Kassa, Timothy M. Nowatzki, Amit Sethi, Jian-Zhou Zhao, Scott Diehn, Virginia Crane, Gary Sandahl, Rongjin Guan, Brad Poland, Claudia Perez Ortega, Mark E. Nelson, Weiping Xie, Lu Liu, Gusui Wu

**Affiliations:** 1 DuPont Pioneer, Johnston, IA 50131 USA; 2 DuPont Pioneer, Hayward, CA 94545 USA; 3 Nexomics Biosciences, Bordentown, NJ 08505 USA

## Abstract

Crops expressing *Bacillus thuringiensis* (*Bt*)-derived insecticidal protein genes have been commercially available for over 15 years and are providing significant value to growers. However, there remains the need for alternative insecticidal actives due to emerging insect resistance to certain *Bt* proteins. A screen of bacterial strains led to the discovery of a two-component insecticidal protein named AfIP-1A/1B from an *Alcaligenes faecalis* strain. This protein shows selectivity against coleopteran insects including western corn rootworm (WCR). Transgenic maize plants expressing *AfIP-1A/1B* demonstrate strong protection from rootworm injury. Surprisingly, although little sequence similarity exists to known insecticidal proteins, efficacy tests using WCR populations resistant to two different Cry proteins show that AfIP-1A/1B and mCry3A differ in their mode of action while AfIP-1A/1B and the binary Cry34Ab1/Cry35Ab1 protein share a similar mode. These findings are supported by results of competitive binding assays and the similarity of the x-ray structure of AfIP-1A to Cry34Ab1. Our work indicates that insecticidal proteins obtained from a non-*Bt* bacterial source can be useful for developing genetically modified crops and can function similarly to familiar proteins from *Bt*.

## Introduction

Western corn rootworm (*Diabrotica virgifera virgifera* LeConte, WCR) and related *Diabrotica* species are serious insect pests of maize (*Zea mays*) production in North America and increasingly also in Europe^1, 2^. The economic impact of this pest is considerable with damage estimates resulting from yield loss and treatment costs to U.S. farmers alone exceeding $1 billion annually^3^. Genetically engineered crops developed with genes of the *Bacillus thuringiensis* (*Bt*) insecticidal crystal (Cry) proteins have been commercialized for the control of WCR. Maize crops with these *Bt* proteins are planted widely providing substantial benefits to farmers and the environment by protecting yield potential and reducing the need for conventional insecticide applications^4^. *Bt* genes used for WCR control in commercialized maize products belong to two Cry protein families: Cry3 (Cry3Bb1, mCry3A and eCry3A.1Ab) and Cry34/35 (Cry34Ab1/Cry35Ab1)^5^. Recent reports of lowered WCR susceptibility to these Cry proteins^6–10^ reflect the emergence of insect resistance and underscore the need for useful alternative proteins that are active against WCR. One such protein is IPD072Aa from *Pseudomonas chlororaphis* ^11^ which demonstrates that non-Bt bacteria, even those that are Gram-negative can be sources of insecticidal proteins useful for transgenic trait development. However, to date, it is not known whether such proteins from non-*Bt* bacteria might share a similar mechanism (i. e., are cross-resistant) with *Bt* Cry proteins.

Here we report the discovery of a two-component WCR-active protein, AfIP-1A/1B, from the Gram-negative bacterium *Alcaligenes faecalis*. Greenhouse and field tests demonstrate that transgenic maize plants expressing *AfIP-1A/1B* show high levels of protection against WCR root feeding. While the amino acid sequences of these proteins bear little similarity to known corn rootworm-active proteins, cross-resistance evaluations, competitive binding studies and comparison of protein structures indicate that *AfIP-1A/1B* kills WCR through a mechanism that is similar to Cry34Ab1/Cry35Ab1. Importantly, our findings show that insecticidal proteins derived from a non-*Bacillus* source can have properties similar to those of *Bt* Cry proteins.

## Results

### Identification of insecticidal AfIP-1A and AfIP-1B proteins

Cell lysates of bacterial isolates were screened for activity against WCR using an artificial diet-based bioassay. Samples resulting in mortality or severe stunting of larval growth were studied further. Activity was observed from strain DDMC-P4G7 that was identified as an *Alcaligenes faecalis* based on its 16 S rRNA gene sequence. An association of *A. faecalis* with insect pathogenicity has been reported previously: *A. faecalis* strain MOR02, isolated from nematodes, was found in larval cadavers of *Galleria mellonella* and subsequently shown to be toxic to *G. mellonella* larvae^12, 13^.

The bioactivity, against WCR, of fractions from DDMC-P4G7 lysates separated by column chromatography guided the development of a multi-step purification scheme. This process led to the isolation of two proteins which only when combined had potent activity (Supplementary Information). When analyzed by denaturing PAGE gels the apparent molecular weight of the smaller protein, designated AfIP-1A, was ~17 kDa and that of the larger AfIP-1B protein was ~76 kDa.

The amino acid sequences of AfIP-1A and AfIP-1B were determined by a combination of N-terminal sequencing, LC-MS/MS analysis and whole genome sequence analysis. Peptide sequences generated by Edman sequencing and LC-MS/MS analysis were searched against open reading frames (ORFs) predicted from DDMC-P4G7 genomic sequence. The peptides aligned perfectly to two adjacent ORFs which were separated by 11 base pairs and appear to form a single operon. *AfIP-1A* represents a 441 nucleotide gene that encodes the AfIP-1A protein of 146 amino acids with a deduced molecular mass of about 16.1 kDa and predicted pI of 4.8. A Shine Dalgarno ribosomal binding site sequence (AGGA) occurs seven nucleotides upstream of the ATG start codon of *AfIP-1A*. *AfIP-1B* has 2,112 nucleotides and encodes the AfIP-1B protein with 703 amino acids, a deduced mass of 76.6 kDa and a predicted pI of 4.5.

PFAM analysis^14^ showed that AfIP-1A has features of the aegerolysin family of proteins (PFAM06355; score of 127 bits and an E-Value of 3e^−31^). Aegerolysins have been described from diverse phyla and share low isoelectric points, similar molecular mass (15–17 kDa) and exhibit broad pH stability. These proteins are predominantly β-structured and some have cell membrane-disturbing activity^15^. Cry34Ab1 (~14 kDa) is also an aegerolysin-like protein which together with Cry35Ab1 causes toxicity to WCR via pore formation in midgut epithelia^16, 17^. AfIP-1A has 20.7% sequence identity and 32.2% similarity to Cry34Ab1. Analysis of AfIP-1B did not reveal a relationship to families currently in the PFAM database.

### Evaluation of activity, potency and selectivity

The coding sequences for AfIP-1A and AfIP-1B were cloned with or without the native stop codon removed and with a 6x-histidine tag into the pET24 vector (Novagen) and separately transformed into *E. coli*. Cell lysates of cultures of all transformations were inactive against WCR in diet assays when each protein was assayed alone but proved insecticidal when both proteins were present.

Insecticidal activity bioassay screens were conducted with purified C-His-tagged AfIP-1A and AfIP-1B. Feeding assays for Coleoptera were performed with neonates placed on an artificial diet containing the proteins. A dilution series with AfIP-1A and AfIP-1B at equal mass ratio starting from 250 ppm of each was used to determine the LC_50_ (concentration for 50% mortality) and IC_50_ (concentration for 50% growth inhibition) against WCR and two other coleopteran pests of maize - northern corn rootworm (*D. barberi* Smith & Lawrence, NCR) and southern corn rootworm (*D. undecimpunctata* Howardi, SCR). The total numbers of dead and stunted larvae (>60% reduction in size compared to control larvae) were used to calculate the IC_50_^11^. High potency against WCR and NCR was observed while SCR was the least sensitive of these *Diabrotica* (Table 1). AfIP1A/1B with each protein at the high dose of 300 ppm resulted in no significant mortality of spotted lady beetle (*Coleomegilla maculata* DeGeer) larvae during a 7 day exposure suggesting selectivity among Coleoptera (data not shown). There was no optimal AfIP-1A/1B ratio for WCR activity when the two components were combined in various proportions. Severe stunting or death was observed when WCR larvae were exposed to 2 to 128 ppm AfIP-1A in the presence of 0.13 to 128 ppm AfIP-1B (Supplementary Table S1). Growth stunting was consistently observed at or above 625 ppm for AflP-1A alone and at or above 3000 ppm for AflP-1B alone.

**Table 1:** Potency of AfIP-1A/1B against *Diabrotica* corn pests in artificial diet bioassay.

Insect^a^	Activity, ppm (95% confidence limits)	
	LC_50_	IC_50_
WCR	30.4 (19.6–43.3)	11.5 (6.9–15.7)
NCR	11.2 (3.0–16.9)	3.3 (2.2–4.5)
SCR	>240^b^	66.7 (2.4–186.2)

AfIP-1A and AfIP-1B were assayed at a 1:1 ratio with values representing combined mass of both components. ^a^WCR, western corn rootworm (*Diabrotica virgifera virgifera)*, NCR, northern corn rootworm (*D. barberi*), SCR, southern corn rootworm (*D. undecimpunctata)*. ^b^12.5% mortality at 240 ppm.

Little or no toxicity was observed in artificial diet assays with AfIP-1A/1B against a number of lepidopteran pests of maize and soybean. There was no effect on European corn borer (*Ostrinia nubilalis* Hűbner), corn earworm (*Helicoverpa zea* Boddie), fall army worm (*Spodoptera frugiperda* JE Smith), or black cutworm (*Agrotis ipsilon* Hufnagel) when both proteins were combined, each at 1000 ppm. Stunting of soybean looper (*Chrysodeixis includens* Walker) and velvet bean caterpillar (*Anticarsia gemmatalis* Hűbner) was observed, but only when a combination of at least 125 ppm of each protein was tested.

### Homologs to AfIP-1A and AfIP-1B

Public and internal sequence databases were explored for proteins with homology to AfIP-1A and AfIP-1B using BLAST searches^18^. Recent *A. faecalis* sequence additions to the National Center for Biotechnology Information database, WP_051316251.1 and WP_052159390.1 with 100% and 97% identity, respectively to AfIP-1A and WP_060185089, WP_035271678.1 and WP_026483257.1 with 96%, 97% and 99% identity to AfIP-1B were noted. WP_026483257.1 is C-terminally truncated by 66 residues with respect to Af1P-1B. These accessions are annotated as hypothetical proteins. Lysates of a selection of different *A. faecalis* acquisitions from the American Type Culture Collection (ATCC®) (Manassas, VA) and the USDA Agricultural Research Service (NRRL) Culture Collection (Peoria, IL) were evaluated for insecticidal activity. Lysates of strains ATCC 15246, ATCC 19209, ATCC 43161, ATCC 49677, USDA B-2542, USDA B-2162 and USDA B-41076 were active against WCR and their genomic DNA was sequenced. There was very little sequence divergence of the amino acid sequences of the AfIP-1A and AfIP-1B homologs from these strains. They showed >96% identity to the respective proteins from DDMC-P4G7 (Supplementary Table S2).

### AfIP-1A/1B protect genetically modified maize from corn rootworm

To test if these proteins can provide root protection, the expression vector ZmAfIP1A/1B was constructed for *Agrobacterium*-mediated maize transformation with *AfIP-1A* and *AfIP-1B*. The ZmAfIP1A/1B transformation vector included a first transgene cassette comprising the root-specific *Sorghum bicolor* RCc3 promoter (gi|478556190|emb|JB072168.1) linked to *AflP-1A* and a second transgene cassette which included the constitutive promoter of the maize ubiquitin (*Ubi-1*) gene^19^ linked to *AflP-1B*. Root injury was assessed for T0 transgenic plants subjected to WCR egg infestations in a greenhouse. Negative (non-transgenic) and positive control plants (containing event DAS-59122-7 that expresses Cry34Ab1/Cry35Ab1 *Bt* proteins) were assayed concurrently. Root feeding was visually assessed using the Iowa State University 0-3 node-injury scale^20^. All of the AfIP-1A/AfIP-1B events generated with ZmAfIP1A/1B showed excellent protection against root injury. Efficacy was comparable to the positive control plants (Supplementary Fig. S1). A selection of homozygous ZmAfIP1A/1B events was advanced for seed increase to allow multi-location field testing. For the field evaluation, second-generation (T2) hybrid maize plants derived from four selected ZmAfIP1A/1B events were tested. The locations and dates of key plot activities at each are reported in Supplementary Table S3. Observations in the plot areas indicated the predominant species present at the locations was WCR. The Rochelle, IL location was also classified as a “Cry3Bb1 problem field” based on substantial root injury (mean ± SE node-injury score of 2.18 ± 0.22, *n* = 20) to a commercial hybrid corn line (MON88017) expressing only Cry3Bb1, which was planted in a separate comparative trial and exposed to natural infestations during the same season.

**Figure 1:**
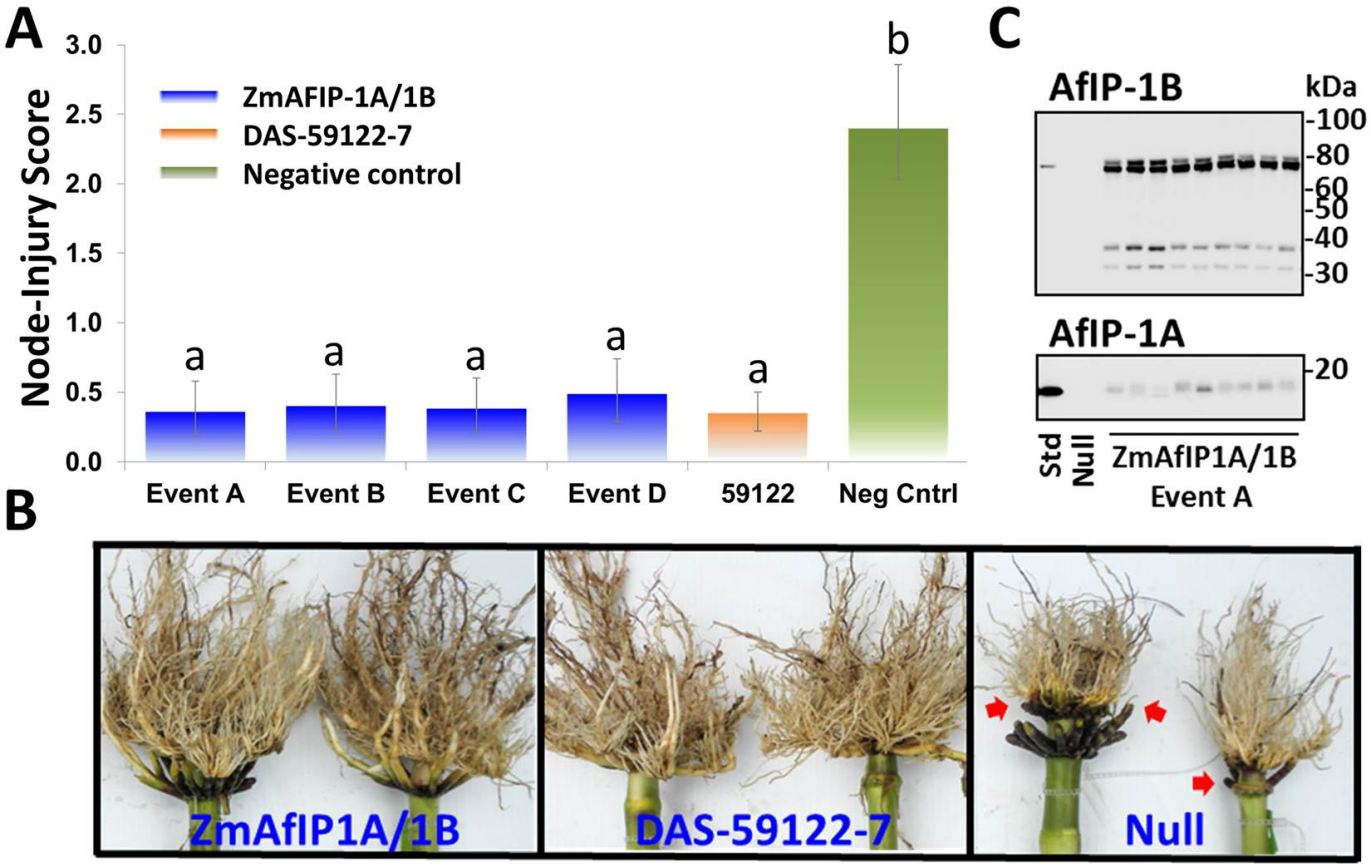
Protection of T2 generation transgenic maize accumulating AfIP-1A/1B in field tests against WCR. (**A**) Comparison of node-injury scores (root ratings) among treatments across three field locations with high corn rootworm pressure in 2012. Injury from corn rootworm larval feeding was visually assessed using the Iowa State 0–3 Node-Injury Scale^20^. Estimated node-injury ratings followed by the same letter were not significantly different. A difference was considered statistically significant if the *p*-value of the difference was less than 0.05. (**B**) Root protection of a subset of the AfIP-1A/1B plants showing efficacy similar to the commercial DAS-59122-7 control while the non-transgenic negative control reveals significant feeding injury (red arrows). (**C**) Western analysis of AfIP-1A and AfIP-1B accumulation in roots of nine siblings from Event A.

Rootworm larval feeding was classified as high at all three testing locations as indicated by the estimated node-injury ratings to the negative control plants which exceeded 2.00 (Supplementary Table S4). Across the three locations, events expressing *AfIP-1A/1B* provided excellent root protection (Fig. 1). The estimated node-injury rating for plants from all four experimental events was not significantly different from the root protection provided by event DAS-59122-7, and all transgenic treatments provided significant protection compared to the negative control (Fig. 1A). Single-location analyses are provided in Supplementary Table S4. Results from the Rochelle, IL location indicated a low likelihood of cross-resistance between AfIP-1A/1B and the Cry3Bb1 protein expressed by event MON88017. At Rochelle, injury to all four ZmAflP1A/1B events was not significantly different from DAS-59122-7 (Supplementary Table S4), and all 4 events had estimated node-injury scores much lower than the injury observed on the commercial MON88017 line reported above. This shows that experimental events with ZmAfIP1A/1B should provide robust efficacy against corn rootworm populations that have developed resistance to the Cry3Bb1 protein^6^. Western blot analysis confirmed accumulation of AfIP-1A and AfIP-1B in roots of field-grown plants. Some *in planta* processing of AF1P-1A and AfIP-1B was observed (Fig. 1C).

### Midgut tissue binding experiments

**Figure 2:**
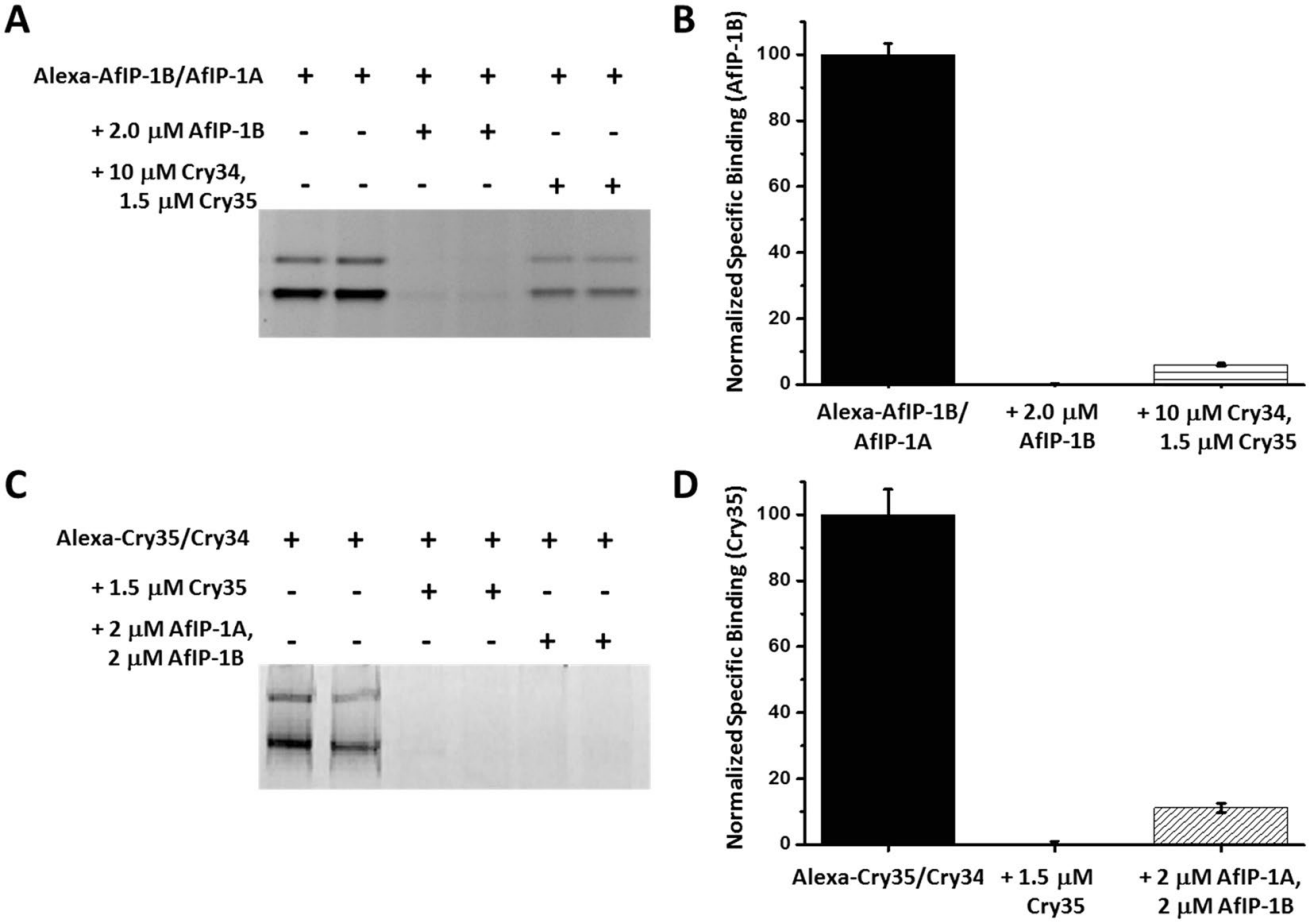
Shared binding sites between AfIP-1A/AfIP-1B and Cry34/35 on BBMVs from WCR midgut tissue. (**A**) Image of in-gel fluorescence after incubation of BBMVs (10 µg) with Alexa-AfIP-1B (5 nM) with AfIP-1A (10 nM), in the absence or presence of an excess of Cry34/35 or AfIP-1B. (**C**) Image of gel representing reciprocal heterologous competition assay of Alexa-Cry35Ab1 (0.07 µM) with Cry34Ab1 (10 µM), in the absence or presence of an excess of Cry34/35 or AfIP-1B. Normalized specific binding of AfIP-1B (**B**) and Cry35Ab1 (**D**) based on optical densitometry of gel images represented in (**A**) and (**C**) after subtraction of nonspecific binding (see Supplementary Information). The data presented are the average and SEM of 3 experiments consisting of 2 or 3 determinations each.

Binding assays involving brush border membrane vesicles (BBMVs) derived from insect midguts provide an established molecular tool to assess the binding site or receptor for engagement of insecticidal proteins and can reveal if two proteins share a common step in their mode of action. Survival of third instar WCR on DAS-59122-7 compared to on non-transgenic maize is reduced^21^. WCR BBMVs of third instars are commonly used for binding tests and specifically bind Cry3Aa and Cry34Ab1/Cry35Ab1^22, 23^. Heterologous competition assays were conducted to determine if AfIP-1A/1B shared midgut binding sites with Cry34/35 or Cry3A (using Cry3A variant IP3-H9^24^). Shared binding sites would indicate the potential for cross-resistance between these proteins. Previous work showed a lack of competition between binding of Cry3Aa and Cry34/35 to WCR midgut BBMVs indicating different target sites utilized for these *Bt*-derived actives in their mode of action pathways^23^. Similar to what has been reported for the binding properties of Cry34/35^23^, empirical testing revealed that the binding of AfIP-1B required the presence of AfIP-1A (data not shown; manuscript in preparation). To test for shared binding sites of AfIP-1A/AfIP-1B with Cry34/Cry35, BBMVs were incubated with Alexa-labeled AfIP-1B (5 nM) along with unlabeled AfIP-1A (10 nM) in binding buffer in the absence and presence of a mixture of an excess of Cry34/35. Complete displacement of specific AfIP-1B binding was observed in the presence of Cry34/Cry35 (Fig. 2A and B). When reciprocal heterologous competition experiments were performed using Alexa-labeled Cry35Ab1 (70 nM) along with Cry34Ab1 (10 µM) in the absence and presence of an excess of unlabeled AfIP-1A/1B, nearly complete elimination of specific binding of labeled Cry35Ab1 was observed (Fig. 2C and D). This confirmed that AfIP-1A/1B and Cry34/35 share binding sites on WCR midgut BBMVs indicating a potential for cross-resistance between these proteins. In contrast, there was no evidence of shared binding sites between Af1P-1A/1B and Cry3 (Supplementary Fig. S2).

### Cross-resistance analysis confirms shared mode of action with Cry34/35 but not mCry3A

**Figure 3:**
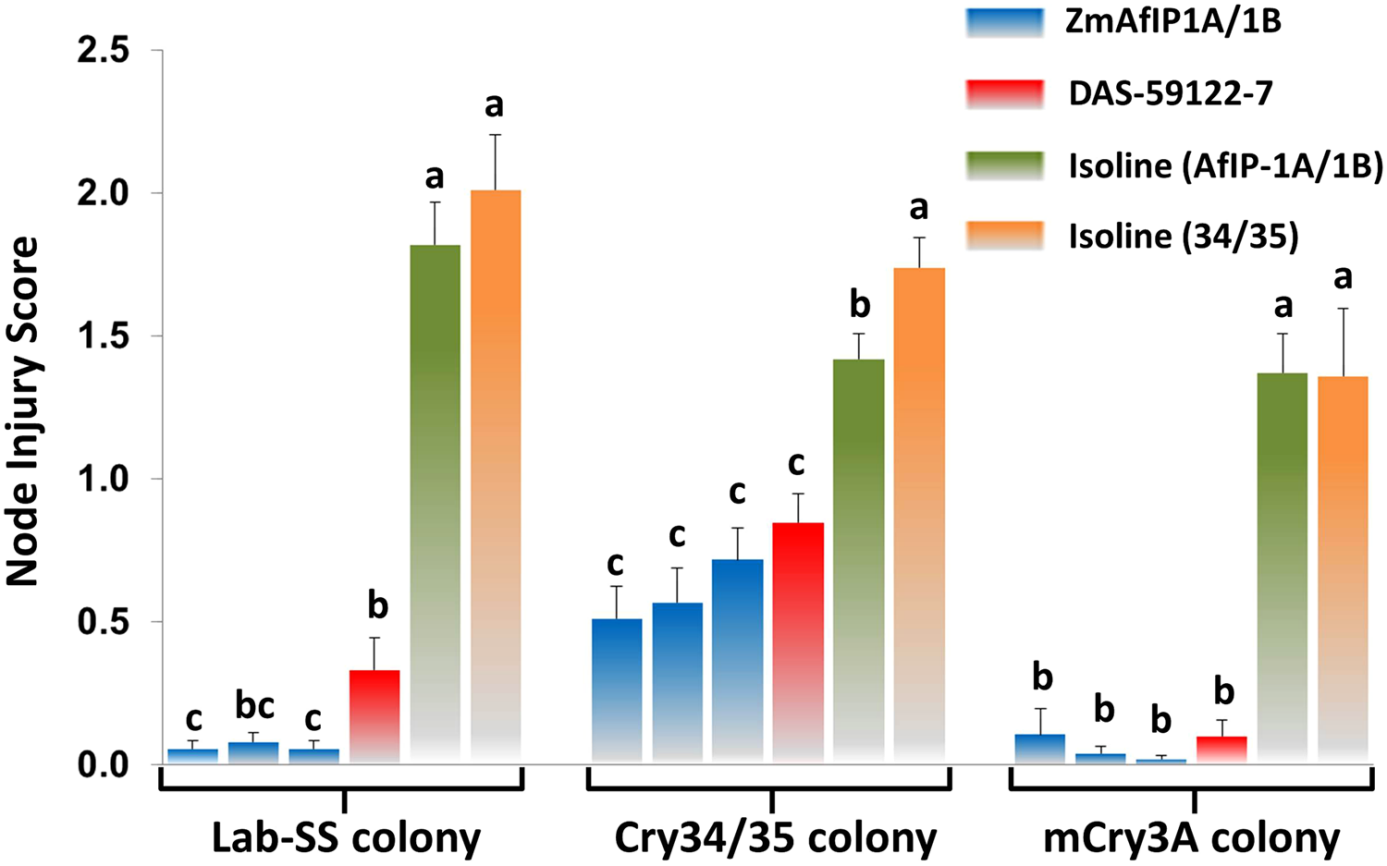
Laboratory-selected WCR confirm similar mode of action of AfIP-1A/1B to Cry34/35 but not to mCry3A. Injury from larval feeding to AfIP-1A/1B maize events by Cry34/35- and mCry3A- selected WCR populations was visually assessed using the 0–3 Iowa State node-injury scale^20^. Means and 95% confidence intervals followed by different letters within insect populations differed significantly (P ≤ 0.05) by using a linear mixed model.

Previous studies demonstrated cross-resistance of field-evolved WCR resistant to Cry3Bb1 to maize expressing *mCry3A* but not Cry34/35 plants^6, 7^. We used laboratory-generated WCR colonies selected for resistance to mCry3A^22^ or Cry34/35^25^ in assays adapted from Nowatzki *et al*.^26^ to assess cross-resistance to AfIP-1A/1B. This involved infesting transgenic mCry3A, Cry34/35, and AfIP-1A/1B maize seedlings with mCry3A- or Cry34/35-selected as well as unselected WCR neonates to evaluate impact on root injury to ZmAfIP1A/1B events. Non-transgenic maize isolines served as negative controls. Consistent with greenhouse and field testing results when compared to isoline maize, ZmAfIP1A/1B events displayed significant protection against feeding by unselected WCR (Fig. 3). However, Cry34/35-selected WCR caused feeding injury to roots of ZmAfIP1A/1B events that was at a similar level as observed with the Cry34/35 expressing event DAS-59122-7. WCR selected for resistance to mCry3A caused as little damage to roots of ZmAfIP1A/1B events as to DAS-59122-7. Thus based on binding assay studies and results with selected WCR colonies there was evidence for cross-resistance and a similar mode of action between Cry34/35 and AfIP-1A/1B but not between mCry3A and AfIP-1A/1B.

### Crystal structure of AfIP-1A

**Figure 4:**
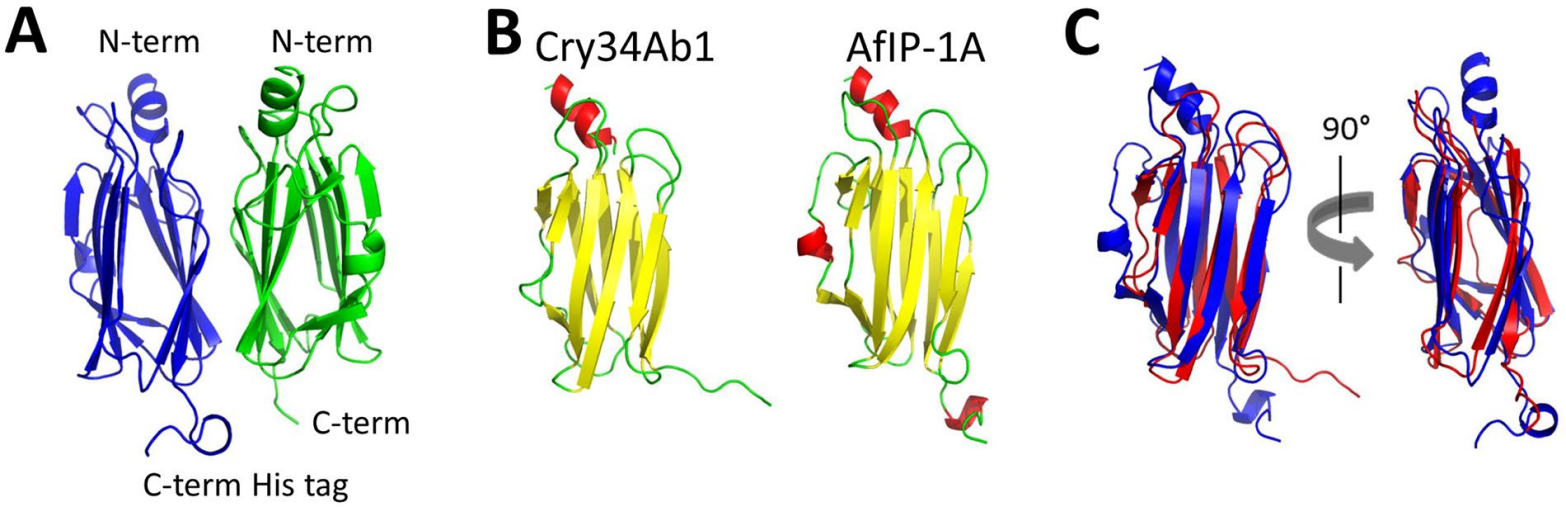
Structure model of AfIP-1A(I20M, T135M) compared to Cry34Ab1 (**A**) X-ray crystal structure of AfIP-1A (PDB ID: 5V3S). Each monomer in the dimer is distinguished by color. (**B**) Structures of Cry34Ab1 (PDB ID: 4JJOX) and AfIP-1A monomer. (**C**) Overlay of Cry34Ab1 (red) with Af1P-1A (blue).

Even though AfIP-1A/1B share little primary amino acid sequence identity to Cry34/35, the cross-resistance and midgut receptor binding results suggest some structural similarity between these protein pairs. We were able to determine structure of AfIP-1A(I20M, T135M), an insecticidal variant of AfIP-1A, to 1.8 Å resolution by x-ray crystallography (Supplementary Fig. S3). In the P2_1_ crystal form the structure of AfIP-1A is a dimer with monomers consisting of 11 beta strands with one short α-helix at the N-terminus (Fig. 4A). The overall fold of the AfIP-1A monomer is a β-sandwich similar to the actinoporin-like pore forming toxins^27^. The N-terminal 8 residues in both monomers are missing from the model due to the lack of electron density in this region, indicative of a highly flexible N-terminal polypeptide. Three surface loops, residues 77–82, 103–109 and 128–131 are also highly mobile with weak or missing electron density. The monomer structures are virtually identical except for the above 3 surface loops and the flexible N-terminal polypeptide segment. Superposition of the two monomers on Cα atoms gave an RMSD of 0.0906 Å (calculated with LSQKAB^28^) over 118 residues. Further description of the crystallographic dimer is available in the “Supplementary Information”.

### AfIP-1A structural comparison to Cry34Ab1

We investigated the structural relationships of AfIP-1A and AfIP-1B to the X-ray crystal structures of Cry34Ab1 (4JOX)^29^. The structure of Cry34Ab1 was superimposed onto the 1.8 Å refined structure of AfIP-1A using Superpose from the CCP4 package^30^. The superimposed structures reveal a high degree of structural similarity between Cry34Ab1 and AfIP-1A (Fig. 4C). The RMS deviation between main chain atoms of the β-sandwich (105 residues) is 1.92 Å. The major structural divergence is observed within the region spanning AfIP-1A Pro46-Ile62 and Cry34Ab1 Thr33-Asn37. Cry34Ab1 has a simple four amino acid coiled region connecting β3 and β4 whereas AfIP-1A contains a three residue turn, five residue β strand, and five residue α helix in the structurally equivalent region (Fig. 4C). The remaining connecting loops between β strands in the AfIP-1A structure adopt a similar confirmation to those in Cry34Ab1 except for the final glycine-rich connecting loop (residue 128-131) between β10 and β11. This loop deviates from that of Cry34Ab1, but this region also has weak electron density. As a result the structural differences here may not be significant. Overall, the structure of AfIP-1A is quite similar to that of Cry34Ab1 providing some explanation for a similar mode of action between AfIP-1A and Cry34Ab1.

### Relationship between AfIP-1B and Cry35Ab1

Given the results from the competitive-binding assays indicating similarity of mode-of-action between AfIP-1A/1B and Cry34/35 and the structural similarity between AfIP-1A and Cry34Ab1, we attempted to probe the existence of any relationship between AfIP-1B and Cry35Ab1. It is important to note that AfIP-1B (703 aa) is significantly larger compared to Cry35Ab1 (385 aa). We were unable to detect any relationship between the sequences of the two molecules or between AfIP-1B and any other sequence in the Protein Data Bank^31^ using sequence or profile alignment methods^18, 32^. Interestingly, some of the members of the aegerolysin family, to which AfIP-1A appears to belong, have binary interacting partners that belong to the Membrane Attack Complex Perforin (MACPF) family^33^. However, we were unable to identify any significant relationship between AfIP-1B and the MACPF family using current sequence/profile alignment methods. Furthermore, no hits were found with HMM-HMM (hidden Markov model) comparisons^34^.

## Discussion

Emerging resistance of insects to transgenic insect control traits based on *Bt* proteins highlights the importance of discovering novel insecticidal proteins that may offer additional modes of action that can substitute or complement existing trait genes. In this report we describe the identification of a two-component insecticidal protein, AfIP-1A/1B, from the Gram-negative *A. faecalis*. This protein shows selective activity against coleopteran pest insects including WCR in artificial diet assays. In transgenic maize the expression of *AfIP-1A/1B* results in plants with healthy roots that are protected from WCR injury in both greenhouse and field tests. This demonstrates that non-*Bt* bacteria can be sources of potent insecticidal proteins. This notion is supported by the recent report of a novel coleopteran-selective insecticidal protein from a *Pseudomonas* species^11^. Assays with WCR colonies selected in the laboratory for resistance to the three-domain *Bt* mCry3A protein or the binary-active Cry34/35 indicate that AfIP-1A/1B has a mode of action that is distinct from mCry3A but is similar to that of Cry34/Cry35. This was confirmed by heterologous competition assays showing that AfIP-1A/1B and Cry34/35 share the same binding sites in WCR midgut membranes. Structural comparison of AfIP-1A and Cry34Ab1 shows that even though there is only ~20% sequence identity between the two sequences, their crystal structures reveal a strikingly similar aegerolysin-like folding pattern. Key elements related to Cry34Ab1/35Ab1 insecticidal activity have been described: a combination of Cry34Ab1 and Cry35Ab1 has been reported to bind specifically to sites on WCR midgut membranes^23^ and results in disruption of WCR midgut epithelium^17^ presumably following ion channel formation^16^. Our cross-resistance data and the structural similarity demonstrate that insecticidal proteins from non-*Bt* bacteria can exert insect toxicity with a similar mode of action as insecticidal *Bt* Cry proteins. On the other hand, the distinct mode of action of AfIP-1A/1B from that of Cry3A also demonstrates that non-*Bt* insecticidal proteins have the potential to be essential new components for insect trait development. Furthermore, these data show that mode of action analyses for any new insecticidal protein needs to extend beyond source organisms and protein sequence similarities.

## Methods

Detailed experimental protocols related to greenhouse and field testing of transgenic plants are provided in Supplementary Methods. Also presented therein are protocols used to perform binding assays with WCR midgut BBMVs and the methods used to determine the structure of AfIP-1A.

### Organisms

*A. faecalis* DDMC-P4G7 is a strain from a DuPont collection of bacterial isolates. Strains ATCC 15246, ATCC 19209, ATCC 43161 and ATCC 49677 were purchased from the American Type Culture Collection and B-2162, B-2542, and B-41076 were from the United States Department of Agriculture Agricultural Research Service Culture Collection.

The WCR colony resistant to mCry3A was developed by selections on mCry3A-transgenic maize plants as described elsewhere^22^. The Cry34Ab1/Cry35Ab1-resistant WCR colony was developed by selections on maize containing event DAS-59122-7^25^.

### Artificial diet insect assays

Rootworm assays were conducted by mixing 15 µl test sample with 65 µl of a modified Diabrotica diet (Frontier Agricultural Sciences, Newark, DE) in each well of a 96-well plate and then adding neonate corn rootworm as described previously^11, 22^. Assays with *C. maculata* followed previously described protocols^35^ except that test proteins were not replaced during the duration of the 7 day assay. For assays against Lepidoptera 25 µl of test proteins were topically applied to 100 µl of a Lepidoptera-specific artificial diet (Southland Products Inc., Lake Village, Arkansas) and allowed to dry before 2 to 3 neonate larvae were placed in each well. Eight wells per sample were used for Coleoptera assays and 4 for Lepidoptera for initial screening of insecticidal activities. For LC_50_ and IC_50_ determinations the total sample size at each concentration was 32 wells. Assays were run at 25 °C for 3–4 days and then visually scored for insect mortality and stunting of larval growth (>60% reduction in size compared to control larvae). Mortality or growth inhibition was scored based on the least affected individual for each well. The data for each bioassay were analyzed by the PROBIT procedure in SAS software (Version 9.4, SAS Institute, Cary, NC)^22^.

### DDMC-P4G7 strain identification and genome sequencing

For species identification of the strain, genomic DNA was extracted using the GenElute Bacterial Genomic DNA kit (Sigma). The 16 S ribosomal sequences were generated by polymerase chain reaction using the HF advantage PCR kit (Clontech) and the 16 S conserved PCR primers AGAGTTTGATCCTGGCTCAG (16 S FOR) and ACGGCTACCTTGTTACGACTT (16 S REV). The 16 S rRNA gene sequence was used as the query sequence for a FastA search (Wisconsin Package Version 9.0, Genetics Computer Group, Madison, Wis.) of GenBank for similar sequences.

For genome sequencing of DDMC-P4G7 genomic DNA was prepared according to a library construction protocol developed by Illumina® and sequenced using the Illumina® Genome Analyzer IIx. The nucleic acid contig sequences were assembled and open reading frames generated as described previously^11^.

### Purification and identification of AfIP-1A and AfIP-1B

The cell pellet of an overnight culture from a single colony of DDMC-P4G7 grown in Luria broth was lysed using a TS-series cell disruptor (Constant Systems Inc.). The extract was clarified and proteins that precipitated with 40% (NH_4_)_2_SO_4_ removed by centrifugation. The (NH_4_)_2_SO_4_ concentration was raised to 80% saturation and the precipitated proteins collected by centrifugation. The resulting pellet was dissolved in 20 mM MOPS, pH 7.1, 10 mM NaCl, and desalted into the same buffer using a GE HiPrep 26/10 (GE Healthcare) desalting column. The eluate was loaded onto a 20 ml Ceramic HyperD® Q anion exchange column (Pall). A linear 7.5 column volume (CV) gradient from 10 to 300 mM NaCl in 20 mM MOPS, pH 7.1, was applied. Fractions eluted from the anion exchange column with WCR activity were concentrated with 10 kDa molecular weight cutoff centrifugal concentrators (Sartorius Stedim), desalted into 5 mM sodium phosphate, pH 6.8 and loaded onto a 22 ml Type 1 Hydroxy Apatite column (Bio-Rad), and a 15 CV gradient to 150 mM sodium phosphate, pH 6.8 was applied. Fractions with WCR-activity were concentrated and loaded onto a Superdex® 200 (GE Healthcare) column equilibrated in 100 mM ammonium bicarbonate. Mixing of two components or fraction pools was found to be required to maintain activity against WCR indicating that more than one protein may be required. Each of the identified WCR-active component pools were then diluted 1:1 with water and loaded onto a 0.8 ml ProSwift® SAX column (Dionex) that was equilibrated in 20 mM MES, pH 6.2 and a 60 CV gradient from 20 to 220 mM NaCl in 20 mM MES, pH 6.2 was applied. Fractions were once again assayed for activity against WCR in the presence or absence of the second component. The WCR-active components were thereby further purified resulting in single bands on Coomassie®-stained LDS electrophoresis gels. To identify the sequences of AfIP-1A and AfIP-1B proteins in gel bands, standard protocols for mass spectrometry (MS) and N-terminal sequencing^11^ were used. The resulting amino acid sequences were BLAST searched against an in-house database that included all bacterial protein sequences from NCBI non-redundant database (nr) and in-house protein sequences, including ORF sequences generated from the genomic sequence of strain DDMC-P4G7.

### AfIP-1A/1B gene cloning

The AfIP-1A and AfIP-1B coding sequences were used to design the following primers to clone the genes: For *AfIP-1A*, GCTGAGGACTTACATATGACTGC (Orf101FOR) and CTTCTATGTCCAGGATCCTCTCCCTTAGG (Orf101REV) and for *AfIP-1B* GGAGAAACATATGGACATAGAAGCTAAATCC (Orf105FOR) and GGAGGATCCCTGAGTTTCAGGCC (Orf105REV) were used. The *AfIP-1A* and *AfIP-1B* genes were PCR amplified and cloned into a pET24 vector (Novagen®) with or without a C-terminal translation of a 6x-histidine tag and transformed into *E. coli* cells. Recombinant proteins of AfIP-1A and AfIP-1B lysates were affinity purified over a Talon™ resin column (ThermoScientific). The His-tagged proteins were eluted with 150 mM imidazole in phosphate buffered saline (PBS). After buffer exchange to PBS using PD-10 desalting columns, lysates were assayed against WCR.

### Maize expression vectors and transformation

Transformation vectors containing molecular stacks of *AfIP-1A* and *AfIP-1B* cDNAs were generated. In construct ZmAfIP1A/1B the root-specific promoter Sb-RCc3^36^ drove expression of the *AfIP-1A*, and a tandem array of the GZ-W64A, UBQ14 and IN2-1 terminators^37^ was cloned 3′ of the gene to terminate transcription and to buffer expression of the downstream cassette containing *AflP-1B* ^38^. The promoter, 5′ untranslated exon and first intron of maize ubiquitin *ubi-1* ^39^ and the NOS terminator^40^ were used to regulate *AflP-1B* expression. ZmAfIP1A/1B was assembled via a Gateway®-based homologous recombination (Invitrogen^TM^) in which the individual expression cassettes were cloned into entry vectors and recombined with a destination co-integrate vector based on pSB1 and pSB11^41–43^ to form a superbinary transformation vector. This destination vector also contained the plant selectable marker gene *phosphomannose isomerase* ^44^ driven by the maize ubiquitin 1 promoter and its intron, and the *moPAT* gene for glufosinate herbicide based selection^45^ driven by the rice actin promoter^46^. The selection cassettes were separated by the CZ19B1 terminator^44^. ZmAfIP1A/1B was introduced into *Agrobacterium tumefaciens* strain LBA4404, and the resulting transformants were used to infect immature embryos of the commercial maize elite-inbred line PHR03 using the protocols of Cho *et al*.^45^ to regenerate transgenic seedlings. T0 maize plantlets expressing ZmAfIP1A/1B were transferred to soil and evaluated in greenhouse-based WCR assays and then backcrossed with PHR03 to generate T1 progeny.

### Root protection of AfIP-1A/1B maize against selected WCR colonies

An on-plant so-called Root trainer assay described previously^11, 25^ was used to assess injury potential of WCR colonies. A total of 18 different treatments were tested involving six seed types – three AfIP-1A/1B expressing events from construct ZmAfIP1A/1B, the event DAS-59122-7 expressing Cry34/35, and two non-transgenic isolines that served as negative controls – each for AfIP-1A/1B and DAS-59122-7, and WCR laboratory populations that were unselected or mCry3A-, or Cry34/35-selected. The experimental design included three rounds of the 18 treatments arranged in a randomized complete block design with 10 replicates of each treatment in each round. Each root trainer container had two plants as subsamples and formed an experimental unit. Statistical analyses were conducted using SAS software, Version 9.3 (SAS Institute Inc., Cary, NC) to compare node-injury scores between treatments. A square root transformation was performed for the raw data of node-injury score before statistical analyses as it improved model goodness-of-fit in terms of satisfying normality and homogenous variance assumptions. A linear mixed model was used to fit the transformed data, and the restricted maximum likelihood estimation method was used to estimate treatment means (known as least squares means). Pair-wise statistical comparisons between treatments were conducted with Tukey’s multiple adjustments, among 6 seed types within each insect type. The means and 95% confidence intervals were then back-transformed to the original data scale for reporting purposes. The experimental design included 12 treatments (3 insect populations x 4 seed types) arranged in a randomized complete block design with 4 replicates of each treatment. Each container was an experimental unit. Statistical analyses were conducted using SAS software, Version 9.3 (SAS Institute Inc., Cary, NC, USA) to compare body size and instar distribution between the treatments.

### Data deposition

The sequences reported in this paper have been deposited in the Gen-Bank of the National Center for Biotechnology Information under the accession numbers KU495732, KU495733, KX550422-KX550435). The atomic coordinates of AfIP-1A have been deposited at the Protein Data Bank, www.pdb.org (PDB ID code 5V3S).

### Distribution of the Materials

The materials reported in this paper may be subject to third party ownership and/or to governmental regulations. Availability of materials described in this manuscript to academic investigators for non-commercial research purposes under an applicable material transfer agreement will be subject to the requisite permission from any third-party owners of all or parts of the material and to governmental regulation considerations. Obtaining permission from third parties will be the responsibility of the requestor. A patent, US9475847(B2), entitled “Insecticidal Proteins and Methods for Their Use” has been issued.

## Electronic supplementary material

Supplementary Information
