# Supplementary material for: An Alcaligenes strain emulates Bacillus thuringiensis producing a binary protein that kills corn rootworm through a mechanism similar to Cry34Ab1/Cry35Ab1
Source: Sci Rep. 2017 Jun 8;7:3063. doi: 10.1038/s41598-017-03544-9 (PMC5465095; doi:10.1038/s41598-017-03544-9)
Supplement: Supplementary file 1 — Supplementary Information [file 41598_2017_3544_MOESM1_ESM.pdf]

Supplementary Information

An *Alcaligenes* strain emulates *Bacillus thuringiensis* producing a binary protein that kills corn rootworm through a mechanism similar to Cry34Ab1/Cry35Ab1

Nasser Yalpani[1*], Dan Altier[1], Jennifer Barry[1], Adane Kassa[1], Timothy M. Nowatzki[1], Amit Sethi[1], Jian-Zhou Zhao[1], Scott Diehn[1], Virginia Crane[1], Gary Sandahl[1], Rongjin Guan[3], Brad Poland[1], Claudia Pérez Ortega[1], Mark E. Nelson[1], Weiping Xie[2], Lu Liu[2] & Gusui Wu[1]

[1]DuPont Pioneer, Johnston, IA 50131 USA; [2]DuPont Pioneer, Hayward, CA 94545 USA; [3]Nexomics Biosciences, Bordentown, NJ 08505 USA.

Corresponding author: Nasser Yalpani, DuPont Pioneer, 7300 NW 62nd Ave., Johnston IA 50131-1004, U.S.A.
Telephone: 515-535-4391
e-mail: nasser.yalpani@pioneer.com

Supplementary Methods

AfIP-1A and AfIP-1B homologs from *A. faecalis* accessions. Strains purchased from the American Type Culture Collection and the USDA ARS NRRL Culture Collection were cultured, lysed and screened for activity against WCR. Their genome sequence was generated as described above. The DNA sequences of homologs of AfIP-1A and AfIP-1B that were identified have been deposited in the GenBank of the National Center for Biotechnology Information under the accession numbers listed in Supplementary Table S2.

Greenhouse testing of transgenic maize. T0 events were infested with 800 WCR eggs per plant at the V3 to V4 stage (3 to 4 collared leaves) and root injury visually assessed at ~V7 using the Iowa State 0-3 node-injury scale[1].

Field testing transgenic maize. Second-generation (T2) hybrid maize seed derived from construct ZmAfIP1A/1B, with four independent transformation events, was tested at three U.S. locations in 2012. Field trials were conducted on land that contained late-planted conventional maize the previous season

to attract corn rootworm beetles for egg laying and to enhance natural infestations. Plots at all locations were also manually infested with 750 wild-type WCR eggs per plant (French Agricultural Research, Inc., Lamberton, MN) using mechanical infesters when plants were between growth stages V2 and V4.

The experimental unit was a single-row plot of corn 5.3 m in length and a row spacing of 76.2 cm. Treatments were arranged in a randomized complete block experimental design and replicated 3 times at each location. Treatments included four events from construct ZmAfIP1A/1B, 2 entries of the commercial event DAS-59122-7 as the positive control, and two non-transgenic negative control entries. All treatments were tested in the same hybrid background. A seed treatment containing the fungicidal active ingredients fludioxonil and metalaxyl, and the insecticide, thiamethoxam, at a rate of 0.25 mg a.i./kernel (Cruiser® 250; Syngenta Crop Protection, Inc., Greensboro, NC) was applied to seeds in all treatments. This is the labeled rate for control of certain secondary insect pests of corn but does not provide control of corn rootworm.

The four experimental events from ZmAfIP1A/1B were sprayed with Ignite® 280SL (24.5% glufosinate, Syngenta Crop Protection Inc., Greensboro, NC) at a rate of 1.6 L/ha when plants were between growth stages V2 and V5 to remove any plants not containing the events of interest. Additionally, plants were leaf sampled prior to root evaluation and analyzed for copy number of the transgenes of interest. Only plants containing single copies of the *AfIP-1A* and *AfIP-1B* genes were included in the statistical analysis.

Root injury was evaluated by digging a sub-sample of 5 roots per plot, washing the root systems clean of soil, and then visually assessing the amount of corn rootworm larval injury using the Iowa State 0-3 node-injury scale[1]. The testing locations, dates of key activities, and levels of corn rootworm larval feeding pressure are shown in Supplementary Table S3.

A linear mixed model was applied to model node-injury scores across locations. To meet a model assumption of normality and equal variance, square root transformation was applied to node-injury scores for data analysis. Data for square-root transformed node-injury score (Y_{ijmnks}) of location $(L)_i$, replication $(R)_j$, construct $(P)_m$, event $(E)_n$, plot $(K)_k$ and plant s, were modeled as a function of an overall mean μ, factors for location, location by replication, treatment, event nested within treatment, location by treatment, location by event nested within treatment, plot within each location $(K/L)_{ik}$ and a residual within each location $(\varepsilon/L)_{ijmnks}$. The model can be specified as:

$$Y_{ijmnks} = \mu + \underline{L_i} + \underline{(L \times R)_{ij}} + P_m + (P \times E)_{mn} + \underline{(L \times P)_{im}} + \underline{(L \times P \times E)_{imn}} + \underline{(K/L)_{ik}} + \underline{(\varepsilon/L)_{ijmnks}}$$

where treatment and event nested within treatment were treated as fixed effects, and all the other effects were treated as independent normally distributed random variables with means of zero. Results across locations were back-transformed to original scales and reported in Fig. 1. An additional by-location analysis using a linear mixed model was conducted to examine node-injury scores for each location. Data for square-root transformed node-injury score (Y_{imnks}) of replication $(R)_i$, treatment $(P)_m$, event $(E)_n$, plot $(K)_k$ and plant *s*, were modeled as a function of an overall mean μ, factors for replication, treatment, event nested within treatment, plot and a residual ε_{imnks}. The model can be specified as:

$$Y_{imnks} = \mu + \underline{R_i} + P_m + (P \times E)_{mn} + \underline{K_k} + \underline{\varepsilon_{imnks}}$$

where treatment and event nested within treatment were treated as fixed effects, and all the other effects were treated as independent normally distributed random variables with means of zero. In both analyses, *F*-tests were used to assess significance for fixed effects. *T*-tests using standard errors from the model were conducted to compare treatment (construct and event) effects. Differences were considered statistically significant if the *P*-value of the difference was less than 0.05. Results by location were back-transformed to original scales and reported in Supplementary Table S4. All data analysis and comparisons were made in ASReml 3.0 (VSN International, Hemel Hempstead, UK, 2009).

Binding assays with WCR midgut brush border membrane vesicles (BBMV). Midguts were harvested from maize-fed third instar larvae and used for BBMV preparations for binding assays as described previously[2]. To track binding, proteins were labeled with Alexafluor 488® (Thermo Scientific) according to manufacturer's recommendations. To simulate the natural processing of the proteins by WCR gut enzymes, AfIP-1A/1B Cry34/35 were processed as follows: Full-length AfIP-1A (1-2 mg/ml) and AfIP-1B (1-2 mg/ml) were incubated with agarose-immobilized TPCK-treated trypsin (Thermo Scientific) at a 1:2 (v:v) ratio of protein:trypsin in PBS buffer, pH 7.4, containing 0.1% Tween 20, in Handee-spin columns (Pierce) at 37 °C for 2 h. Processing of AfIP-1A results in an N-terminally truncated product that runs close to 16 kDa in denaturing gels. Trypsinization of AfIP-1B results in the appearance of a ~40 kDa N-terminal fragment and a ~37 kDa C-terminal fragment of the protein on denaturing gels (as is also observed following incubation in WCR gut fluid). Trypsinized AfIP-1A/1B retains insecticidal activity against WCR (data not shown). Processing of full-length Cry34Ab1 by soluble TLCK-chymotrypsin (Sigma Aldrich) was performed in processing buffer (50 mM NaCl, 20 mM Tris, pH 8.5) using a 100:1 (w:w) ratio of protein:chymotrypsin, at 37 °C for 15 min. Processing of full-length Cry35Ab1 by soluble TLCK-chymotrypsin was performed in the same buffer as Cry34Ab1, but using a ratio of 50:1 (w:w) protein:chymotrypsin, at 25 °C overnight. Reactions were stopped using 1 mM phenylmethanesulfonyl fluoride (G-Biosciences). Proteins were dialyzed into binding buffer prior to binding assessments. The

processing of Cry34Ab1 results in an N-terminally truncated form that runs close to 15 kDa in denaturing gels. Purification of processed proteins was not needed to achieve specific binding. Prior to binding experiments, proteins were quantified by gel densitometry following Simply Blue® (Thermo Scientific) staining of SDS-PAGE resolved samples that included BSA as standards. Specific binding of AfIP-1A/AfIP-1B and competition binding assays were conducted essentially as described previously[2]. We determined that specific binding of Alexa-labeled AfIP-1B to WCR BBMVs requires the presence of AfIP-1A (data not shown). Specificity of binding was demonstrated by elimination of Alexa-labeled AfIP-1B binding in the presence of saturating concentrations of unlabeled AfIP-1B (see Fig. 2A and C). Competition against binding of modified Cry3A protein, IP-3H9[2,3], was characterized in a PBS Binding Buffer (PBS, pH 7.2, 0.1% Tween20®, protease inhibitor cocktail [Roche Diagnostics]). Competition of AfIP-1A/1B against Cry34/Cry35 was assessed in a Bis-Tris Binding Buffer (20 mM Bis-Tris, pH 6, 100 mM KCl, 0.1% Tween20®, protease inhibitor cocktail) as it provided favorable solubility conditions for the proteins. Signals from triplicate experiments each consisting of 2 or 3 determinations were averaged and reported as normalized specific binding which was calculated by subtracting the nonspecific binding signal from all densitometry values for that experiment. The non-specific binding signal was defined as the fluorescence remaining in the presence of saturating concentrations of homologous competitor for each labeled protein. When specific binding was reflected by more than one protein band, densitometry values for the most intense band were used to quantify binding.

Expression and purification of AfIP-1A(I20M, T135M). Multi-wavelength anomalous diffraction (MAD) provides a means to solve the phase problem in protein crystallography. The incorporation of seleno-methionine (SeMet) residues into proteins is a common practice. However, wild-type AfIP-1A protein has only an N-terminal Met. We generated the double mutant I20M and T135M by site-directed mutagenesis and expressed C-term-6x-His tagged AfIP-1A(I20M, T135M) in BL21 Gold and used IMAC to purify the protein. The purified double mutant showed WCR activity that was comparable to the tagged wild-type protein in diet assays (Supplementary Fig. S3). For SeMet incorporation C-term-6x-His tagged AfIP-1A(I20M, T135M) was expressed in T7 Express Crystal cells (New England BioLabs®). Starter cultures were grown overnight in Hi-Def Azure media (Teknova), 1% glucose, 15 µg/ml kanamycin, 50 µg/ml L-methionine at 200 rpm until the OD reached ~0.8 AU, the cells were pelleted and resuspended in the same media lacking Met and grown for an additional 2.5 h at 37 °C. L-seleno-methionine was then added to a final 66 µg/ml and the temperature adjusted to 16 °C. After 30 min IPTG was added to 0.5 mM and the cultures were grown at 150 rpm overnight. Cells were lysed in 2xPBS containing EDTA-free proteinase inhibitors (Roche) using a TS-series cell disruptor (Constant Systems Inc.) and the lysate

clarified by centrifugation. The supernatant was loaded onto a 100 ml Talon Superflow IMAC column (GE Healthcare) equilibrated in 2xPBS. The column was washed extensively with 2xPBS, 6 mM imidazole and protein eluted with 2xPBS, 150 mM imidazole. The eluate was further purified using a 20 ml SuperQ-5PW anion exchange column (Tosoh Bioscience LLC) equilibrated in 20 mM MOPS, pH 7.1, and eluted with a 12 CV 0 to 300 mM NaCl gradient in this buffer. Further purification was achieved by chromatography with a 26/60 HiLoad S200 Prep Grade (GE Healthcare) column in PBS, 1 mM DTT. Eluted AfIP-1A was lyophilized and brought to a final concentration of 16.7 mg/ml in PBS, 10 mM Tris, 1 mM DTT. MS analysis of the purified protein revealed complete SeMet incorporation [Expected mass of labeled AfIP-1A(I20M, T135M) if N-terminal methionine is absent is 17,173.7 Da; the observed mass was 17,173.8 Da]. Static light scattering data were collected on a miniDAWN (TREOS) light scattering instrument (Wyatt Technology) coupled with an analytical gel filtration column (Protein KW-802.5, Shodex, Japan) with AfIP-1A at 16.15 mg/ml. All measurements were performed in 10 mM Tris-HCl, pH 8, 100 mM NaCl, 1 mM TCEP at room temperature and with a flow rate of 0.5 ml/min. Data analysis using the ASTRA software package (Wyatt Technology) showed that under these conditions AfIP-1A is >98% monodisperse with an estimated mass 32.62 kDa.

Crystallization and Structure determination of AfIP-1A. Initial high-throughput crystallization screening was carried out by micro batch method using screening kits both from Hampton Research and self-made covering 1,536 conditions, with the AfIP-1A protein concentration at 7.98 mg/ml. The experiments were set up at 4 °C for the first week and then transferred to 22 °C from the second week. The screening identified a number of crystallization conditions which yielded protein crystals, and then these conditions were further optimized manually by fine tuning the concentration of both protein solution and precipitants as well as the pH. Diffraction quality crystals were obtained in 0.1 M Hepes, pH 7.0, 10% PEG 1000 and 0.1 M sodium malonate. Seven diffraction datasets were collected on six crystals at the National Light Source, Brookhaven National Laboratory. The space group was determined to be $P2_1$ monoclinic with unit cell dimensions of a=59.29 Å, b=34.42 Å, c=71.44 Å, $\alpha = \gamma = 90.0^o$, $\beta = 96.54^o$. The asymmetric unit contains two molecules, each with two Se atoms. Therefore there are four Se atoms in the asymmetric unit whose anomalous light scattering was used to obtain initial phase information by SAD (single-wavelength anomalous dispersion/diffraction) method. The *Crank* program from the CCP4 suite (4) was used to locate the Se atom positions (*Crunch2*), calculate initial SAD phases (*BP3*), determine hand (*Solomon*) and improve phases by density modification (*Parrot*). The CCP4 program *Buccaneer* allowed building a partial structure. This was followed by manual model building and adjusting with *Coot*. Structure refinement was carried out using *Refmac5*. The structure was refined

against the 1.8 Å resolution data to final R and R-free factors of 21.51% and 26.76%, respectively (Supplementary Table S7). The overall quality of the structure model is very good (Molprobity and Ramachandran, Supplementary Table S7). Several residues in surface loops have very weak electron densities, therefore it is not possible to define the structure in these regions. The four outliers in the Ramachadran plot, residues 129 and 131, in both molecules are located in a very flexible surface loop. Supplementary Table S8 contains additional structure refinement and validation statistics.

The structure of AfIP-1A is a dimer with monomers consisting of 11 beta strands with one short α-helix at the N-terminus (Fig. 4A). The N-terminal 8 residues in both monomers are missing from the model due to missing electron density in this region, indicative of a highly flexible N-terminal polypeptide. Three surface loops, residues 77-82, 103-109 and 128-131 are also highly mobile with weak or missing electron density. The monomer structures are virtually identical except for the above three surface loops and the flexible N-terminal polypeptide segment. Superposition of the two monomers on Cα atoms gave an RMSD of 0.0906 Å (calculated with *LSQKAB*) over 118 residues.

The dimer interface buries a large molecular surface (1224.7 Å^2) as calculated by *Areaimol*. The interface is largely hydrophobic with 52 van der Waal contacts and two side chain hydrogen bonds. The side chain OE2 atom Glu66 for an intermolecular H-bond with the side chain ND2 atom of Asn28 from the other monomer. Besides residues Asn28 and Glu66, other residues that comprise the dimer interface are Thr23, Phe25, Val27, Lys126, Asn136, Ile137, Phe138, Thr140, and Val142.

Supplementary References

1. Oleson, J. D., Park, Y. L., Nowatzki, T. M. & Tollefson, J. J. Node-injury scale to evaluate root injury by corn rootworms (Coleoptera: Chrysomelidae). *J. Econ. Entomol.* **98,** 1-8 (2005).
2. Bermudez, E., Cong, R., Hou, J. T. & Yamamoto, T. Inventors; E.I. DuPont De Nemours and Company, assignee. Synthetic insecticidal proteins active against corn rootworm. US Patent Application 20120210462 A1 (16 August 2012).
3. Zhao, J. Z. *et al.* mCry3A-selected western corn rootworm colony exhibits high resistance and has reduced binding of mCry3A to midgut tissue. *J. Econ. Entomol.* **109,** 1369-1377 (2016).
4. Winn M. D. *et al.* Overview of CCP4 suite and current developments. *Acta Cryst.* **D67,** 235-242 (2011).

AfIP-1B, ppm	AfIP-1A, ppm					
	**128**	**32**	**8**	**2**	**0.5**	**0.13**
**128**	2.7 ±0.5[a]	2.7 ±0.5	2.3 ±0.5	2.2 ±0.4	2.2 ±0.4	2.2 ±0.4
**32**	2.3 ±0.5	2.8 ±0.4	2.3 ±0.5	2.2 ±0.5	2.0 ±0.6	1.8 ±0.4
**8**	2.3 ±0.5	2.5 ±0.5	2.0 ±0.0	2.0 ±0.0	1.7 ±0.5	1.3 ±0.5
**2**	2.7 ±0.5	2.2 ±0.4	2.2 ±0.4	2.2 ±0.4	1.3 ±0.5	1.5 ±0.5
**0.5**	2.2 ±0.4	2.3 ±0.5	2.0 ±0.0	1.5 ±0.5	1.5 ±0.5	0.5 ±0.5
**0.13**	2.0 ±0.0	2.0 ±0.0	2.0 ±0.0	2.0 ±0.0	0.8 ±0.8	1.7 ±0.4

Supplementary Table S1. Impact of dilutions of AfIP-1A and AfIP-1B on inhibition of WCR in diet assay.

[a]Values are WCR inhibition scores: 3 (dead), 2 (severely stunted - little or no growth but alive), 1 (stunted - growth to 2nd instar but not equivalent to controls), or 0 (no activity). Data presented are the mean of 6 replicates per treatment +/- SD. AfIP1A/1B doses with inhibition scores averaging less than 2 are shaded.

Source strain	*AfIP-1A* homolog	% pn Identity to *AfIP-1A* pn	% pp Identity to AfIP-1A pp	*AfIP-1B* homolog	% pn Identity to *AfIP-1B* pn	% pp Identity to AfIP-1B pp
DDMC-P4G7	KU495732	-	-	KU495733	-	-
ATCC 15246	KX550422	100	100	KX550429	99.8	99.7
ATCC 19209	KX550427	96.6	96.6	KX550434	94.3	97.0
ATCC 43161	KX550423	99.5	100	KX550430	99.3	99.4
ATCC 49677	KX550428	91.6	96.6	KX550435	94.4	97.0
USDA B-2162	KX550426	99.5	100	KX550433	99.3	99.4
USDA B-2542	KX550425	99.5	100	KX550432	99.3	99.4
USDA B-41076	KX550424	100	100	KX550431	90.4	99.4

Supplementary Table S2. Genbank accession codes of *AfIP-1A/1B* from *A. faecalis* strains. pn, polynucleotide; pp, polypeptide.

Location	Planting date	Infestation date	Root evaluation date
Mankato, MN	14-May	5-June	20-July
Rochelle, IL	14-May	8-June	16-July
Janesville, WI	8-May	5-June	24-July

Supplementary Table S3. Field testing locations, dates of key activities in 2012.

Location	Treatment[a]	Estimated node-injury score[b]	95% confidence intervals Lower	Upper
Rochelle, IL	Event A	0.10 b	0.01	0.54
	Event B	0.55 b	0.16	1.17
	Event C	0.34 b	0.06	0.84
	Event D	0.70 b	0.25	1.37
	DAS-59122-7	0.34 b	0.12	0.68
	Negative control	2.89 a	2.07	3.84
Janesville, WI	Event A	0.55 b	0.28	0.92
	Event B	0.42 b	0.18	0.76
	Event C	0.49 b	0.24	0.83
	Event D	0.55 b	0.28	0.91
	DAS-59122-7	0.45 b	0.25	0.71
	Negative control	2.24 a	1.77	2.78
Mankato, MN	Event A	0.40 b	0.13	0.81
	Event B	0.26 bc	0.06	0.62
	Event C	0.32 bc	0.09	0.69
	Event D	0.28 bc	0.07	0.64
	DAS-59122-7	0.26 bc	0.09	0.52
	Negative control	2.13 a	1.43	2.99

Supplementary Table S4. Comparison of node-injury scores on back-transformed scale among treatments at 3 field locations in 2012. [a]Events A, B, C, and D were experimental ZmAfIP1A/1B events; only plants confirmed as single-copy for the event of interest were included in the analysis. The commercial event DAS-59122-7 was the positive control and the Negative control contained no events for control of corn rootworm. [b]Injury from corn rootwrom larval feeding was assessed with root ratings on the Iowa State University 0-3 Node-Injury Scale. Within each location, estimated node-injury scores followed by different letters are significantly different ($P < 0.05$).

Location	Source	***dfa***	***F* value**	***P-valueb***
Rochelle, IL	Treatment	3, 21	17.62	<0.01
	Treatment X Event	3, 21.3	1.36	0.28
Janesville, WI	Treatment	3, 20.6	39.43	<0.01
	Treatment X Event	3, 21.7	0.23	0.88
Mankato, MN	Treatment	3, 19.8	23.43	<0.01
	Treatment X Event	3, 20.6	1.261	0.32

Supplementary Table S5. Fixed effects of treatment and treatment x event on square-root transformed node-injury scores from field evaluations at 3 locations in 2012. [a]*df*, numerator degrees of freedom, denominator degrees of freedom. [b]*F*-test considered significant difference if the *P*-value is less than 0.05.

Location	Effect	Estimate	Standard error	Z-ratio[a]
Across locations	Location	0.000	0.006	0.057
	Location X Replication	0.010	0.009	1.129
	Location X Treatment	0.000		N/A
	Location X Event(Treatment)	0.000		N/A
	at(location, Rochelle):Plot	0.085	0.030	2.828
	at(location, Janesville):Plot	0.018	0.009	2.097
	at(location, Mankato):Plot	0.034	0.015	2.306
	at(location, Rochelle):Residual	0.033	0.005	6.326
	at(location, Janesville):Residual	0.033	0.005	6.743
	at(location, Mankato):Residual	0.037	0.006	6.254
Rochelle, IL	Replication	0.000		N/A
	Plot	0.077	0.030	2.585
	Residual	0.033	0.005	6.326
Janesville, WI	Replication	0.018	0.021	0.840
	Plot	0.019	0.009	2.061
	Residual	0.033	0.005	6.743
Mankato, MN	Replication	0.006	0.013	0.445
	Plot	0.042	0.020	2.141
	Residual	0.037	0.006	6.251

Supplementary Table S6. Random effects from the across-location and by-location analyses on square-root transformed node-injury scores from field evaluations at 3 locations in 2012. [a]*Z*-ratio is the ratio between the estimate of the random effect and its own standard error. The effect is considered significantly greater than 0 if *Z*-ratio is greater than 2.

Data collection	Data set 1		Data set 2
Space group	*P2$_1$*		*P2$_1$*
Resolution (Å)	2.0		1.8
Cell dimensions			
a, *b,* c (Å)	59.22, 34.42, 71.38		59.29, 34.42, 71.44
a, *b, g* (°)	90, 96.65, 90		90, 96.54, 90
Wavelength (Å)	0.97869		0.97869
Resolution (Å)	2.0 (2.02-1.99)*		1.80 (1.83-1.80)*
Reflections	19256 (595)		26266 (1238)
R_{merge} (%)	15.2 (20.8)		9.6 (49.6)
Completeness (%)	96.0 (59.7)		97.4 (96.0)
I/sigmal	22.20 (6.71)		14.81 (2.06)
Redundancy	7.1 (3.0)		3.8 (3.8)
Refinement			
Resolution (Å)		1.8	
No. reflections		26255	
R_{work} / R_{tree} (%)		21.51 / 26.76	
No. atoms			
Protein		2258	
Water		272	
B-factors (Å^2)			
Protein		24.32	
Water		30.62	
R.m.s. deviations			
Bond lengths (Å)		0.0179	
Bond angles (°)		1.873	
Molprobity			
Clash score[#]		7.99 (86th percentile[¥])	
Molprobity score[^]		2.06 (58th percentile[¥])	
Ramanchandran plot[Δ]			
Favored		94.31%	
Allowed		99.29%	
Outliers		0.71%	

Supplementary Table S7. AfIP-1A crystallography structure data collection and refinement statistics. *Values in parentheses are for highest-resolution shell. [¥]100th percentile is the best among structures of comparable resolution; 0th percentile is the worst. For clashscore the comparative set of structures was selected in 2004 (N=837, 1.80 Å ± 0.25 Å), for MolProbity score in 2006 (N=11444, 1.80Å ± 0.25 Å). [^]MolProbity score combines the clashscore, rotamer, and Ramachandran evaluations into a single score, normalized to be on the same scale as X-ray resolution. [#]Clashscore is the number of serious steric overlaps (> 0.4 Å) per 1000 atoms. [Δ]Ramachandran analysis was done with Molprobity. The 4 outliers (residues 129 and 131 in both molecules in the asymmetric units) are all in a very flexible surface loop.

A chain				B chain				Distance	
Residue	Position	Atom		Residue	Position	Atom		(Å)	
Thr	23A	OG1	…	Lys	126B	CD	…	3.69	
Phe	25A	CG	…	Thr	140B	CG2	…	3.98	
Phe	25A	CD1	…	Thr	140B	CG2	…	3.77	
Phe	25A	CE1	…	Thr	140B	CB	…	3.88	
			…	Thr	140B	CG2	…	4.01	
Phe	25A	CZ	…	Val	142B	CG2	…	3.69	
Phe	25A	CE2	…	Phe	25B	CE2	…	3.87	
			…	Phe	25B	CD2	…	4.02	
			…	Val	27B	CG2	…	3.86	
Phe	25A	CD2	…	Phe	25B	CE2	…	3.91	
			…	Phe	25B	CD2	…	4.00	
Val	27A	CG1	…	Glu	66B	CD	…	4.08	
			…	Glu	66B	OE2	…	3.32	
Val	27A	CG2	…	Phe	25B	CE2	…	4.02	
Asn	28A	CG	…	Glu	66B	OE2	…	3.41	
Asn	28A	ND2	…	Glu	66B	CD	…	3.57	
			…	Glu	66B	OE2	…	2.53	***
Glu	66A	CG	…	Val	27B	CG1	…	1.10	
Glu	66A	CD	…	Asn	28B	ND2	…	3.46	
			…	Val	142B	CG1	…	3.85	
Glu	66A	OE1	…	Asn	28B	ND2	…	3.45	*
			…	Val	142B	CG1	…	3.27	
Glu	66A	OE2	…	Asn	28B	CG	…	3.69	
			…	Asn	28B	ND2	…	2.97	***
Lys	126A	CB	…	Phe	138B	CE1	…	3.79	
Asn	136A	CB	…	Phe	138B	CE2	…	3.69	
Asn	136A	C	…	Phe	138B	CE2	…	3.89	
Ile	137A	C	…	Phe	138B	CZ	…	4.03	
			…	Phe	138B	CE2	…	3.85	
Phe	138A	CB	…	Phe	138B	CZ	…	3.37	
			…	Phe	138B	CE2	…	3.61	
			…	Phe	138B	CD2	…	3.71	
			…	Phe	138B	CG	…	3.56	
			…	Phe	138B	CD1	…	3.33	
			…	Phe	138B	CE1	…	3.23	
Phe	138A	CG	…	Phe	138B	CB	…	4.00	
			…	Phe	138B	CG	…	3.63	
			…	Phe	138B	CD1	…	3.50	
			…	Phe	138B	CE1	…	3.92	
Phe	138A	CE1	…	Thr	140B	CG2	…	4.01	
Phe	138A	CZ	…	Phe	25B	CB	…	4.02	
			…	Thr	140B	CG2	…	3.77	
Phe	138A	CD2	…	Phe	138B	CB	…	4.01	
			…	Phe	138B	CG	…	3.80	
			…	Phe	138B	CD1	…	3.31	
			…	Phe	138B	CE1	…	3.88	
Thr	140A	CB	…	Phe	25B	CE1	…	3.82	
Thr	140A	CG2	…	Phe	25B	CE1	…	3.87	
			…	Phe	25B	CG	…	3.93	
			…	Phe	25B	CD1	…	3.74	
Val	142A	CG1	…	Glu	66B	CD	…	3.57	
			…	Glu	66B	OE1	…	3.43	
			…	Glu	66B	OE2	…	3.68	
Val	142A	CG2	…	Phe	25B	CZ	…	3.88	

Supplementary Table S8. Interactions between the two monomers of AfIP-1A.

*Close contacts across dimer interface

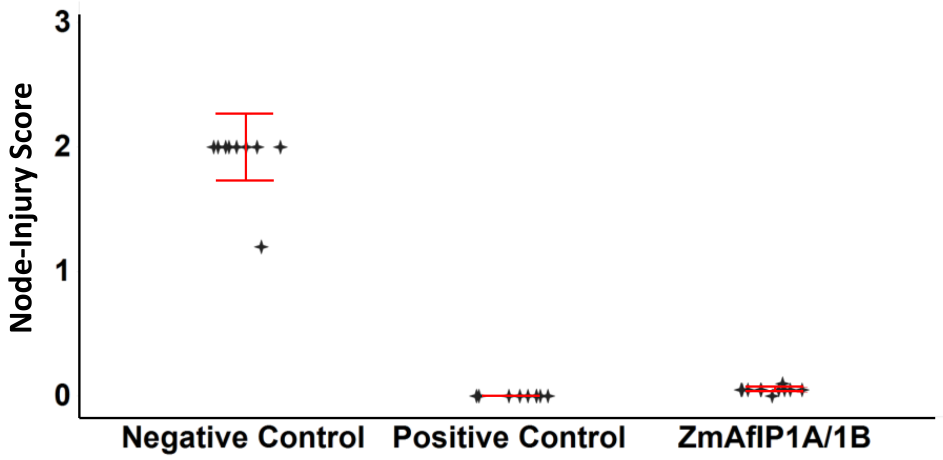

Supplementary Figure S1. Root protection of T0 generation ZmAfIP1A/1B transgenic maize plants against feeding by WCR. Seedlings of 11 independent transformation events expressing single gene copies of *AfIP-1A* and *AfIP-1B* were infested with WCR in a greenhouse at the V3-V4 stage. Root feeding damage was scored at ~V7 as described previously[1] and compared to positive control events expressing *Cry34/35* (n=9) and non-transgenic maize (n=9). Error bars indicate 95% confidence intervals.

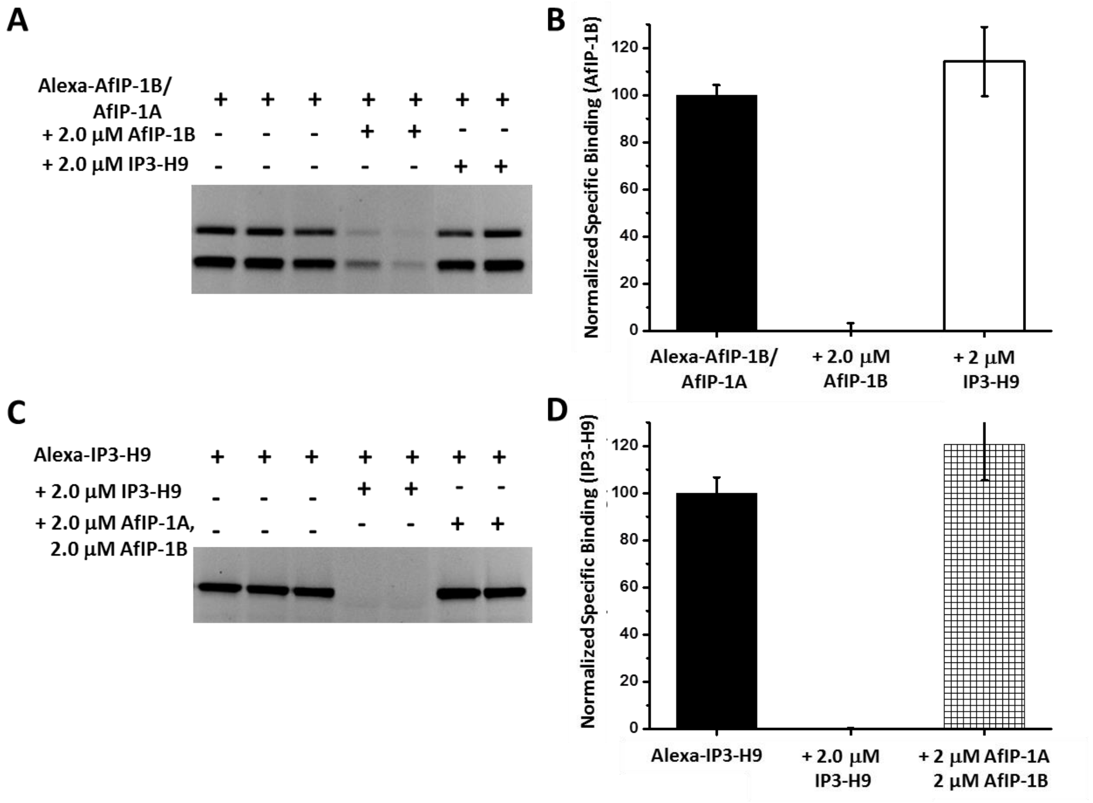

Supplementary Figure S2. Distinct sites of binding of AfIP-1A/1B and Cry3A to BBMV from WCR midgut tissue. (A) In-gel fluorescence after incubation of BBMV (5 µg) with Alexa-AfIP-1B (10 nM) with AfIP-1A (100 nM), in the absence or presence of an excess of Cry3A (IP3-H9) or AfIP-1B. (C) Reciprocal heterologous competition assay against Alexa-IP3-H9 (5 nM) binding in the absence or presence of an excess of IP3-H9 or AfIP-1B. Normalized specific binding of AfIP-1B (B) and IP3-H9 (D) based on optical densitometry of gel images represented in (A) and (B) after subtraction of nonspecific binding as described in SI Materials and Methods. The data presented in bar graphs are the average and SEM of 3 experiments each consisting of 2 or determinations.

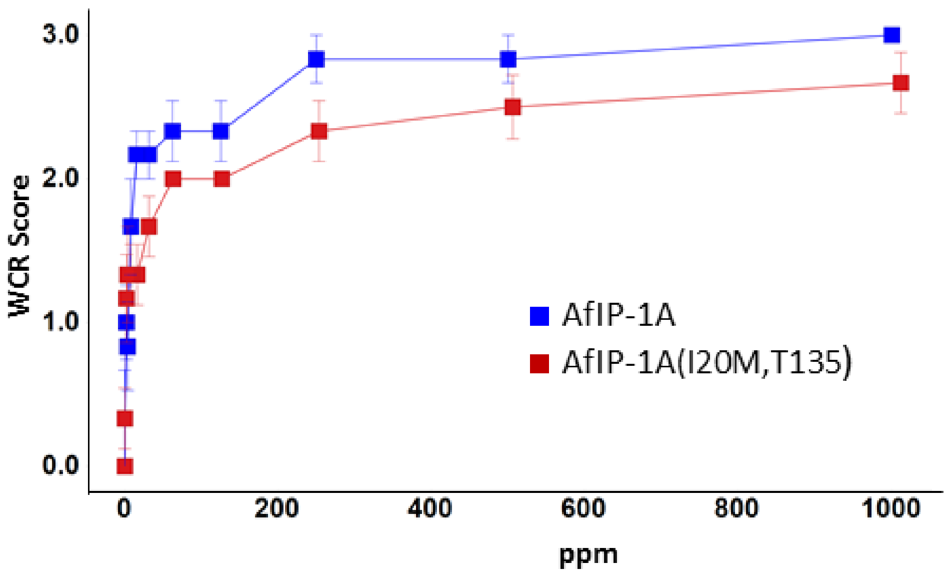

Supplementary Figure S3. Activity of AfIP-1A double mutant against WCR. C-terminally 6xHis-tagged wild-type AfIP-1A and AfIP-1A(I20M, T135M) were used in the diet bioassay in the presence of 100 ppm AfIP-1B. The scores were noted as dead (3), severely stunted (2) (little or no growth but alive), stunted (1) (growth to second instar but not equivalent to controls), or no observed activity (0). The data presented are the average and SEM of 6 replicates.
